# Retrospective Detection of *Ophidiomyces ophidiicola* from Snake Moults Collected in Bieszczady Mountains, Poland

**DOI:** 10.3390/microorganisms12071467

**Published:** 2024-07-19

**Authors:** Daniele Marini, Piotr Szczygieł, Katarzyna Kurek, Matteo Riccardo Di Nicola, Jean-Lou C. M. Dorne, Maria Luisa Marenzoni, Joëlle Rüegg, Stanisław Bury, Łukasz Kiraga

**Affiliations:** 1 Department of Organismal Biology, Evolutionary Biology Centre, Uppsala University, 75236 Uppsala, Sweden; daniele.marini@ebc.uu.se (D.M.); joelle.ruegg@ebc.uu.se (J.R.); 2 Department of Veterinary Medicine, University of Perugia, 06126 Perugia, Italy; marialuisa.marenzoni@unipg.it; 3 Scientific Society of Veterinary Medicine Students, Faculty of Veterinary Medicine, Warsaw University of Life Sciences—SGGW, 02-776 Warsaw, Poland; s202489@sggw.edu.pl; 4 Department of Wildlife Conservation, Institute of Nature Conservation Polish Academy of Science, 31-120 Cracow, Poland; kkurek@iop.krakow.pl; 5 Faculty of Veterinary Medicine, Department of Pathobiology, Pharmacology and Zoological Medicine, Wildlife Health Ghent, Ghent University, 9820 Merelbeke, Belgium; 6 Unit of Dermatology and Cosmetology, IRCCS San Raffaele Hospital, 20132 Milan, Italy; 7 Asociación Herpetológica Española, 28911 Leganés, Spain; 8 Methodology and Scientific Support Unit, European Food Safety Authority (EFSA), 43126 Parma, Italy; jean-lou.dorne@efsa.europa.eu; 9 Department of Comparative Anatomy, Institute of Zoology and Biomedical Research, Jagiellonian University, 30-387 Cracow, Poland; stanislaw.bury@uj.edu.pl; 10 NATRIX Herpetological Association, 52-010 Wrocław, Poland; 11 Division of Pharmacology and Toxicology, Department of Preclinical Sciences, Institute of Veterinary Medicine, Warsaw University of Life Sciences—SGGW, 02-786 Warsaw, Poland; lukasz_kiraga@sggw.edu.pl

**Keywords:** ophidiomycosis, snake fungal disease, SFD, Aesculapian snake, *Zamenis longissimus*, sheds

## Abstract

*Ophidiomyces ophidiicola*, the causative agent of ophidiomycosis, poses a potential threat to wild snakes worldwide. This study aimed to retrospectively investigate the prevalence of *O. ophidiicola* in archived snake moults collected from the San River Valley in the Bieszczady Mountains, Poland, from 2010 to 2012. Using qPCR for *O. ophidiicola* detection and conventional PCR for clade characterisation, we analysed 58 moults and one road-killed specimen of *Zamenis longissimus* and *Natrix natrix*. A novel combination of primers (ITS2L) was used to simultaneously confirm SYBR Green-based qPCR results and perform genotyping. *O. ophidiicola* has been detected from two *Z. longissimus* and one *N. natrix* specimens. The identified clade (I-B) is consistent with those found in wild snakes of eastern Europe and San River Valley, indicating that *O. ophidiicola* has been present in this region for at least a decade. This study underscores the value of historical samples in understanding the long-term presence of pathogens and highlights the potential role of environmental reservoirs in the persistence of *O. ophidiicola*. Our findings are crucial for informing conservation strategies for the endangered Aesculapian snake populations in Poland, emphasising the need for ongoing monitoring and habitat management to mitigate the potential impact of ophidiomycosis.

## 1. Introduction

Fungal pathogens are increasingly recognised as a significant threat to wildlife [1]. These pathogens often have the ability to infect a wide range of hosts and demonstrate environmental resilience [2]. For instance, fungal diseases caused by *Batrachochytrium dendrobatidis*, *B. salamandrivorans*, and *Pseudogymnoascus destructans* frequently result in high mortality rates among amphibians and bats [3,4,5]. Although there is less specific information available on reptile populations, these animals are also at risk to suffer from infectious diseases. Additionally, it is estimated that at least 20% of evaluated reptile species worldwide are endangered [6]. Emerging pathogens, especially taxa from the Onygenales order, pose a global concern for reptiles [7], affecting both veterinary care [7,8] and wild herpetofauna [9,10].

*Ophidiomyces ophidiicola* is a fungal pathogen within the family Onygenaceae, recognised as the causative agent of ophidiomycosis, also referred to as Snake Fungal Disease (SFD) [11]. This pathogen may pose a significant threat to snake populations globally [12]. The taxonomy of *O. ophidiicola* has undergone several revisions and it shares a close phylogenetic relationship with other onygenalean fungi that infect reptiles included in the genera *Nannizziopsis*, *Paranannizziopsis,* and *Emydomyces* [13,14,15]. *O. ophidiicola* is believed to be horizontally transmitted [16] and can persist in soil, particularly in hibernacula (overwintering sites), which may serve as reservoirs for the pathogen [17]. It can grow across a broad range of pH levels and temperatures, and it exhibits tolerance to air dryness and various natural sulphur compounds [18]. Whereas its growth can be inhibited by other soil microbes, it thrives in sterilised soil, suggesting that natural microbial communities play a role in suppressing its proliferation [17]. Infected snakes can transmit the fungus to healthy individuals through direct contact, which is a particular concern in shared environments like hibernacula during brumation [16,19]. Reports indicate postnatal mortality in neonate snakes due to the rapid development of ophidiomycosis, likely transmitted from the dam to the offspring during or after birth [20]. *O. ophidiicola* has been detected in at least 62 snake species across nine families and various continents [12]. It has been linked to significant declines in snake populations, such as the severe decline in *Crotalus horridus* in New Hampshire and high mortality rates in *Sistrurus catenatus* in Illinois [21,22]. The fungus infects snakes through the skin, often without the need for pre-existing wounds or abrasions, although such injuries can increase the likelihood of infection [19]. The infection leads to the development of cutaneous lesions, including regional swelling, scale oedema, discoloration, and crust formation [19]. In severe cases, the infection can become systemic, spreading to internal organs such as the lungs, liver, kidneys, and others, leading to granuloma formation in the tissues [23,24]. Infected snakes may exhibit behavioural and metabolic disorders, notably thermoregulation and behavioural fever [25,26]. These conditions impair predator avoidance by increasing basking behaviour [27]. Furthermore, affected snakes may experience reduced foraging and the suppression of the hypothalamic–pituitary–gonadal axis, influencing reproduction in a sublethal manner [26,28]. Increased frequency of ecdysis may help to clear out the cutaneous infection [26,29]. Three *O. ophidiicola* distinct clades were identified [30,31,32]: Clade I is found exclusively in wild European snakes; Clade II in North American and European wild snakes, and in a Taiwanese wild *Dinodon rufozonatum* and captive snakes worldwide; and Clade III—and related—only in captive snakes and in a wild *Naja atra* from Taiwan. Ladner and colleagues [32] suggest that the fungus likely originated in Eurasia, and that a potential spillover into North America might have occurred via the pet trade or snake translocations. In contrast, Origgi et al. [33] present an alternative scenario wherein Clade II might have been introduced into Europe before the 1950s, since Clade I has not been detected yet outside Europe.

In Europe, *O. ophidiicola* screenings have increasingly been undertaken, resulting in the detection of the pathogen in 13 countries and 10 European wild snake species included in the genera *Coronella*, *Hierophis*, *Natrix*, *Vipera*, and *Zamenis* [29,30,33,34,35,36,37,38,39,40,41,42,43]. Both *O. ophidiicola* Clades I and II were found in free ranging ophidians of Europe [38,41,44], and have occurred here at least since the late 1950s [33]. Although the evidence does not support a definitive conclusion, genotypes associated with more severe disease in North America (Clade II) appear to result in greater disease severity compared to genotypes described in Europe (Clade I—[41]). Additionally, a case of *Zamenis longissimus* mortality is likely associated with *O. ophidiicola* Clade II-D/E in Spain [43]. However, a higher infection prevalence in the continent is best explained by an interaction between host species identity and pathogen clade, suggesting that this relationship varies among different ophidian species [41]. Moreover, in Europe, *O. ophidiicola* appears to exhibit greater tropism towards semi-aquatic snakes, i.e., the *Natrix* genus, and individuals from areas with high human disturbance; in contrast, the sampling season did not show a significant impact on infection prevalence [38,41]. Skin lesions induced by *O. ophidiicola* infection seem to be mostly mildly severe and located in the ventral scales of adult snakes [40]. 

The Aesculapian snake (*Zamenis longissimus*) is distributed from southern Spain to northern Turkey and the eastern Black Sea coast, reaching the Krasnodar region in Russia, Georgia, and Turkey [45]. In Europe, its range coincides with deciduous forests in the temperate zone, with habitat preferences varying geographically. Southern populations inhabit semi-open, slightly damp areas, while northern populations are found in river valleys and increasingly synanthropic environments [45,46,47,48,49,50]. Four isolated areas exist at the northern edge of its range, including two in Germany, one in the Czech Republic, and one in Poland. These populations are considered rare, endangered, and in need of active protection [45,48,49,50,51]. The Polish populations are primarily found in the Bieszczady and Sanocko-Turczańskie Mountains, with sparse occurrences in Beskid Niski Mountains [52,53,54]. However, the populations from Sanocko-Turczańskie Mountains and Beskid Niski Mountains require further research to understand their size and structure. Additionally, it is not known whether the Bieszczady and Sanocko-Turczańskie Mountains populations are connected, especially considering that the Aesculapian snake has a home range of only 3 km [55]. The most studied, abundant, and well-known population in the San River Valley (Otryt Range, San Valley Landscape Park, Bieszczady Mountains) was initially estimated at about 75 individuals in the 1990s, but more recent evaluations place it at approximately 230 snakes [56]. However, differences in research efforts and methods might have underrated the initial estimate. Historically tolerated and even revered by aboriginal communities, the Aesculapian snake faced persecution from new settlers who arrived in Bieszczady in the 1960s, following the 1940s desettlement during Operation Vistula [50,57]. The construction of the Solina Dam on the Rivers San and Solina in the 1960s severely threatened the species by impeding dispersal and contributing to the extinction of northern populations. Landscape changes, including natural woodland succession on former farmland and afforestation projects following World War II, further impacted the species [53]. At its northern range limit, the species shows a strong affinity for anthropogenic habitats such as compost heaps, manure piles, and sawdust heaps, which provide stable temperature and humidity conditions crucial for thermoregulation and successful egg incubation [58,59]. This dependence on human-modified environments is also observed in other reptile species at their northern European range limits [60,61]. The lack of such anthropogenic habitats may significantly influence reproductive success and population structure of Aesculapian snakes [56]. Currently, the main Polish population of the Aesculapian snake in the Bieszczady Mountains is completely isolated, despite the species’ continuous range being 30 km away in Slovakia and Ukraine [52,62]. 

In the 2024 study by Blanvillain and colleagues [41], the prevalence of *O. ophidiicola* in Poland in samples collected from March 2020 to June 2022 was among the lowest recorded in the study, at 2.7% (95% confidence interval [CI]: 0.3%, 5.1%). The study included a comparable sample size to other inspected locations, but the prevalence remained low. Out of 185 samples (58 *Coronella austriaca*; 75 *Natrix natrix*; 11 *Natrix tessellata*; 41 *Zamenis longissimus*) just 5 were found as qPCR-positive to *O. ophidiicola*. Interestingly, all the positives came from the same location, the Bieszczady region, and comprised 4 grass snakes (*N. natrix*: 4/10, 40%) and 1 Aesculapian snake (*Z. longissimus*: 1/41, 2.4%; prevalence for both species: 5/51, 9.8%). The genotype of *O. ophidiicola* belonged to I-B, which is primarily found in wild snakes of eastern Europe [41]. Despite the low prevalence in *Z. longissimus*, the detection of the fungus in the San River Valley raises a great concern for one of the last Polish populations belonging to this species. Therefore, *O. ophidiicola* as a potential threat needs attention given the vulnerability and low abundance of the Aesculapian snake’s isolated population in Bieszczady.

This study aimed to retrospectively investigate *O. ophidiicola* occurrence in archived moults of *Zamenis longissimus* and *Natrix natrix* from the San Valley Landscape Park, Poland, comparing its prevalence to recent findings by Blanvillain et al. [41] and evaluating whether *O. ophidiicola* presence could contribute to the persistently low abundance of Aesculapian snakes in Poland.

## 2. Materials and Methods

### 2.1. Study Species, Study Site, and Moult Collection

Active population monitoring projects for the Aesculapian snake were initiated and conducted by the Institute of Nature Conservation of the Polish Academy of Sciences between 2009 and 2011, extending up to 2014. The research was conducted in compliance with the ethical codes and legislation of the Republic of Poland under scientific permits (DOPozgiz-4200/II-12/1040/09/ed; DOP-OZGIZ.6401.02.3 2011.JRO.1) and ethical permits (39/2008; 29/2010). As part of these initiatives, snake moults were perpetually collected until 2014. 

The primary objective of these projects was to identify the presence of the Aesculapian snake in the Bieszczady and Sanocko-Turczańskie Mountains. The historical occurrence was reviewed [63,64,65] and in order to confirm historical and current information on the Aesculapian snake, the fieldwork was carried out in 2009–2013. The research involved obtaining records of both live and dead individuals during the species’ activity season, as well as collecting moults and interviewing foresters or local people. Information on the snake’s occurrence was gathered until 2015. In addition to these efforts, 51 artificial breeding mounds were set [66]. The mounds were deployed in permanently open areas, such as meadows, wildlife food plots, and deciduous woodlands. Most of the mounds were built in stream and river valleys, near existing villages or ruined buildings in villages depopulated in the 1940s. If it was not possible to build a mound at the planned site where snakes had been observed, it was set up at another spot, but no further away than 1.5 km from the planned locality based on data relating to the size of individual territories and the distances covered by the snake [52,55,62,67]. Concurrently, interviews with locals were conducted, and new sites were successively included. The mounds were monitored for snake colonisation every two weeks from April to October, i.e., when the snakes were active [66]. 

Surveys were carried out between the villages of Zatwarnica and Rajskie in 2009–2013. *Zamenis longissimus* and *N. natrix* sheds were collected during the described fieldwork (see further information in Table 1). The snake moults were placed in paper envelopes and subsequently stored at −20 °C at the Institute of Nature Conservation of the Polish Academy of Sciences (IOP). 

### 2.2. Shed Sampling

In September 2022, 57 moults from 2010 to 2012, and two exceptional samples, a deceased road-killed carcass and a 2004 moult kept previously as a souvenir by a local citizen, were selected for the analysis in this study. *Z. longissimus* moults were prioritised over those of other species (i.e., *N. natrix*). Each moult was singularly extracted from its envelope and examined for gross signs consistent of ophidiomycosis (e.g., brownish crusts, signs of dysecdysis—see Table 1 and Figure S1). 

Three portions of moults (ca. 0.5 × 0.5 cm) were sampled from each specimen. They were systematically excised from cephalic (when possible), dorsal, and ventral scales, and dry-collected in a nucleic acid-free 1.5 mL tube. The dorsal and ventral scale excisions were carried out in distant regions of the trunk. If the moult was showing any gross signs, an extra portion including the lesion was collected. To avoid cross-contamination, the procedure was performed using tweezers and surgical scissors sterilised with ethanol 95% and a Bunsen burner, while surfaces were disinfected with bleach.

### 2.3. Molecular Analysis and Clade Characterisation

*Ophidiomyces ophidiicola* presence was firstly assessed via qPCR as described by Marini et al. [36]. Briefly, DNA was extracted from the moult fractions collected in 1.5 mL tubes by adding 50 μL of PrepMan Ultra Sample Preparation Reagent (ThermoFisher, Carlsbad, CA, USA) and 50 mg of 0.5 mm diameter zirconium oxide beads to the vials. Bullet Blender Storm 24 (Next Advance, Inc., New York, NY, USA) was used to homogenise the samples for 60 s, followed by a centrifugation (30 s at 13,000 RPM), and these steps were repeated twice. Samples were heated for 13 min at 95 °C with a Techne^®^ Dri-Block^®^ DB-2D (Buch & Holm, Herlev, Denmark), cooled for 5 min, and centrifuged again (30 s at 13,000 RPM). Following this, 100 μL of nuclease-free water was added, the samples were centrifuged (30 s at 13,000 RPM), and 50–75 µL of a supernatant was recovered and transferred to 1.5 mL tubes. Subsequently, centrifugation was repeated and finally 30–50 μL of the extract was retrieved. DNA concentration was measured using TECAN Spark^®^ Multimode Microplate Reader (Table S1). The tubes were either stored at −20 °C or directly used. Aliquots with different DNA concentrations were prepared from each DNA extract. For each sample, a dilution of 12.5 ng/μL was made. If amplification results were unsatisfactory for certain samples, additional aliquots at concentrations of 6.25 ng/μL and 25 ng/μL were prepared.

As in Marini et al.ii’s work [36], DNA from each tissue sample was initially tested in triplicates using an SYBR Green-based qPCR for internal transcribed spacer 2 (ITS2—primers designed by Bohuski et al. [68], Table S2) within the ribosomal RNA (rRNA) gene complex of *O. ophidiicola* and for the mitochondrial NADH dehydrogenase subunit 1 (nad1—primers designed by Lorch et al. [69], Table S2) of *O. ophidiicola*. Each 10 µL reaction contained 5 µL of iQ SYBR Green Supermix (Bio-Rad Laboratories Inc., Hercules, CA, USA), 50 ng of DNA in a volume of 4 μL (12.5 ng/µL—original concentrations in Table S1), 0.7 μL of nuclease-free water, and 0.3 μL of a 10 µM suspension of forward and reverse primers. Amplification was performed using a CFX385™ Touch Real-Time PCR Detection System (Bio-Rad Laboratories Inc., Hercules, CA, USA) with the following cycling conditions: 3 min at 95 °C, 40 cycles of 3 s at 95 °C, and 30 s at 60 °C, followed by a melt curve from 65 °C to 95 °C (increment of 0.5 °C and plate read every 5 s). qPCR results, comprising Ct values, melting curves, and the relative fluorescence units (RFUs), were analysed using Bio-Rad CFX Maestro software 1.1 (v.4.1.2) to select positive and suspicious samples to confirm or refute with a further qPCR run for both genomic and mitochondrial targets. The efficiency of the PCR assay, the positive and negative control used, and the RFU analysis in the End-Point mode of the software (Bio-Rad CFX Maestro) are reported by Marini et al. [36]. Subsequently, DNA from each suspected sample was further analysed in triplicate for each dilution (6.25 ng/µL, 12.5 ng/µL, and 25 ng/µL; i.e., 25, 50, and 100 ng of total DNA for each reaction, respectively) to rule out false positive results using the above-mentioned qPCR assay for both ITS2 and nad1 targets. 

**Figure 1:**
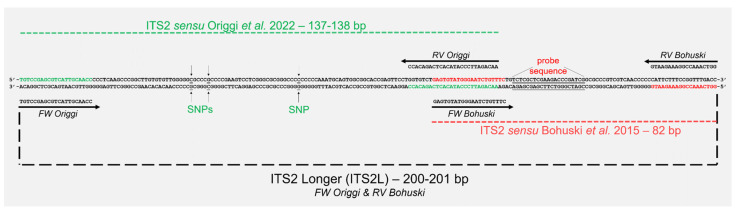
The diagram of the primer sets used to amplify the internal transcribed spacer 2 (ITS2) region within the ribosomal RNA (rRNA) gene complex of *Ophidiomyces ophiidicola*. The forward primer from the Origgi et al. [33] PCR assay (in green) and the reverse primer from the Bohuski et al. [68] qPCR assay (in red) were combined in this study to create the ITS2 Longer (ITS2L) PCR assay. This assay allows for the characterisation of the clade according to Origgi et al. [33] and Blanvillain et al. [41], based on single-nucleotide polymorphisms (SNPs), and corroborates the SYBR Green-based qPCR results as described by Marini et al. [36]. The partial ITS2 used in the image corresponds to a representative sequence of *O. ophidiicola* Clade I (genotype I-B according to Blanvillain et al. [41]).

Further selected samples considered positive were submitted to clade characterisation by amplification via conventional PCR of the most polymorphic fragments (≤200 bp) belonging to ITS2, transcription elongation factor 1 α (TEF), and actin (ACT) genes of *O. ophidiicola* following Origgi et al. [33] (primers in Table S2). Moreover, we used the combination of the ITS2 forward primer by Origgi et al. [33] and the ITS2 reverse primer by Bohuski et al. [68] to provide a longer conventional PCR product whose sequence can (i) confirm the ITS2 SYBR Green-based qPCR *sensu* Marini et al. [36] and (ii) give the most polymorphic fragment of ITS2 *sensu* Origgi et al. [33] and Blanvillain et al. [41] at the same time (see Figure 1 and Table S2). We will refer hereinafter to this primer set as ITS2 Longer (ITS2L).

These genomic fragments were amplified using the PyroMark PCR kit (Qiagen, Hilden, Germany) with the following components: 12.5 µL of PyroMark PCR Master Mix, 7 µL of nuclease-free water, 2.5 µL of CoralLoad Concentrate, 0.5 µL (500 nM) of a forward primer, 0.5 µL (500 nM) of a reverse primer, and 2 µL (50 ng) of a DNA template, for a total of 25 µL per reaction. The PCR conditions were 95 °C for 15 min; 40 cycles of 94 °C for 30 s, 52 °C (TEF) or 55 °C (ACT; ITS2L) or 58 °C (ITS2) for 30 s, and 72 °C for 30 s; and a final extension at 72 °C for 10 min. The products were then separated on a 2% agarose gel for visualisation and size determination under a UV transilluminator.

Bands with expected size were excised from the agarose gel and extracted using the QIAquick^®^ Gel Extraction Kit following the manufacturer’s instructions. Alternatively, after inspecting the qPCR results or the DNA fragments, amplification products from the well were directly purified using the QIAquick^®^ PCR Purification Kit according to the manufacturer’s instructions. Purified PCR products were directly Sanger-sequenced by a commercial service (Macrogen, Amsterdam, The Netherlands) on both strands using the same primers employed for the amplification. The resulting sequences were edited and assembled using BioEdit software (v.7.2.5) and compared with those deposited in GenBank using the Basic Local Alignment Search Tool (BLAST, https://blast.ncbi.nlm.nih.gov/ (accessed on 29 June 2024)—Table S3).

Samples that yielded successful amplification via conventional PCR for clade characterisation and subsequent sequencing were used to construct a maximum likelihood phylogenetic tree using MEGA software (v11.0.13). To construct the phylogenetic trees, we analysed the sequences from our study and a population set of homologous sequences sourced from GenBank and the literature. The sequences included strains from all lineages of *O. ophidiicola* and an outgroup (*Pseudoamauroascus australiensis*—see Table S4). ACT, TEF, and ITS2 sequences were concatenated, MUSCLE-aligned, trimmed, and concatenated again. The Tamura–Nei substitution model was employed, assuming uniform rates across all sites, a complete deletion of gaps, and 3000 bootstrap replications.

Moreover, ITS2 sequences with single-nucleotide polymorphisms (SNPs), able to differentiate *O. ophidiicola* clades according to Blanvillain et al. [41], were aligned with representative *O. ophidiicola* genotype sequences deposited in GenBank using AliView (v1.27) and BLAST to obtain the specific genotype.

### 2.4. Statistical Analysis

To assess the statistical significance of our findings, we utilised the Chi-square test to examine the dependence between categorical variables in the dataset (i.e., *O. ophidiicola* detection against species, month of the year, and gross signs; gross signs against species and period of the year). A logistic regression analysis was applied to explore the relationship between the dependent (*O. ophidiicola* positivity) and independent variables (species, period of the year, and gross signs) to examine whether species, period of the year, and presence of gross signs influenced the likelihood of testing positive for *O. ophidiicola*. A *p*-value of <0.05 was deemed significant. Furthermore, we calculated 95% confidence intervals to estimate the precision of our outcomes. All statistical procedures were performed using Python (v3.12.3).

## 3. Results

A total of 58 moults and a road-killed specimen were collected and analysed in this study. In total, 54 out of 59 samples belonged to *Z. longissimus* (91.5%), while 5/59 (8.5%) belonged to *N. natrix*. 

**Figure 2:**
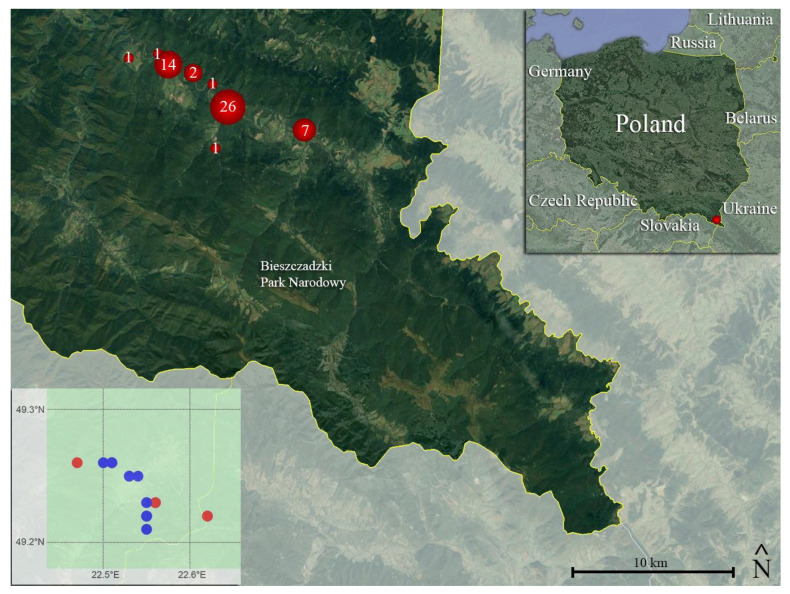
The map of the study area. The figure shows the location and numbers of moults collected in the site (red dots). The dots are roughly proportional to the sample size. Note that the samples represented amount to 53 (i.e., the 5 exuviae lacking precise location/coordinates and the road-killed specimen are not represented in the map—see Table 1). Inset (top right): The location of Bieszczady Mountains in Poland (red dot). Inset (bottom left): The schematic map of the study area representing in red the locations with *O. ophidiicola* detection and in blue the locations with no *O. ophidiicola* detections. Map credit: Google Earth [Data SIO, NO-AA, U.S. Navy, NGA, GEBCO Image Landsat/Copernicus], modified.

Specimens were collected in ten different locations of Bieszczady Mountains (see Figure 2, and Table 1—5 specimens do not have specific toponim or coordinates), with Zatwarnica (22 samples) and Koliba (14 samples) being the most represented sampling localities. 

**Figure 3:**
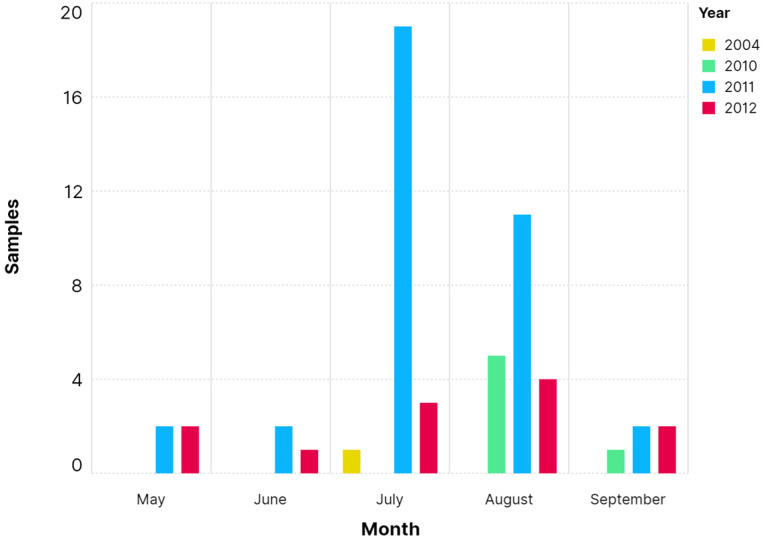
The distribution of sample quantities, with a total of 59 samples, across sampling times from 2004 to 2010. The histogram shows the number of samples collected (*y*-axis) for each sampling time point (*x*-axis), with data grouped by month and year. The graph was made with Scimago Graphica.

Exuviae were collected mostly during the months of July and August in the years 2011 and 2012 (see Figure 3). 

A lower number of specimens were found to have signs consistent with *O. ophidiicola* infection (17/59, 28.8%) compared to those without signs of tegument infection.

At least one of the triplicates from eight samples showed suspicious-to-positive results in the initial runs of SYBR Green-based qPCR, as determined by examining the Ct values, melting curves, and RFUs. Specifically, sample ID 59, 187, and 228 were suspicious-to-positive for both targets; ID 28, 149, 179, and 216 were suspicious only for ITS2, and ID 218 only for nad1. Consequently, these eight samples were subjected to a further qPCR run with the 3-point dilution series of DNA. From this subsequent qPCR amplification, only three samples yielded positive results. Sample ID 149 was positive in three out of nine wells for the ITS2 target, with Ct values ranging from 36.27 to 37.55 and one positive call at an End RFU of 881. Sample ID 218 was positive in two out of nine wells for the nad1 target, with Ct values of 35.97 and 36.43, and positive calls at End RFUs of 1150 and 914. Sample ID 228 was positive in all wells for both ITS2 and nad1 targets. For ITS2, the Ct values ranged from 29.16 to 31.67, with End RFUs ranging from 2094 to 2594. For nad1, the Ct values ranged from 30.09 to 31.88, with End RFUs ranging from 1843 to 3148. The Ct values of the positive control ranged from 26.63 to 24.05 for ITS2 and from 23.82 to 24.27 for nad1. The End RFUs for the positive control ranged from 1735 to 2963 for ITS2 and from 1884 to 2870 for nad1. The melting peaks for the wells of the mentioned samples were between 84 and 84.5 °C for ITS2 (with the positive control at 84.5 °C) and between 73.5 and 74 °C for nad1 (with the positive control at 73.5–74 °C). The amplification and melting curves generated by Bio-Rad CFX Maestro software for these wells are presented in Figure S2. 

Subsequently, DNA from ID 149, 218, and 228 was submitted to conventional PCR for ACT, TEF, ITS2, and ITS2L for clade characterisation. Under the UV transilluminator, only sample 228 showed expected bands for all the targets; they were consistent with the targets’ expected size.

**Figure 4:**
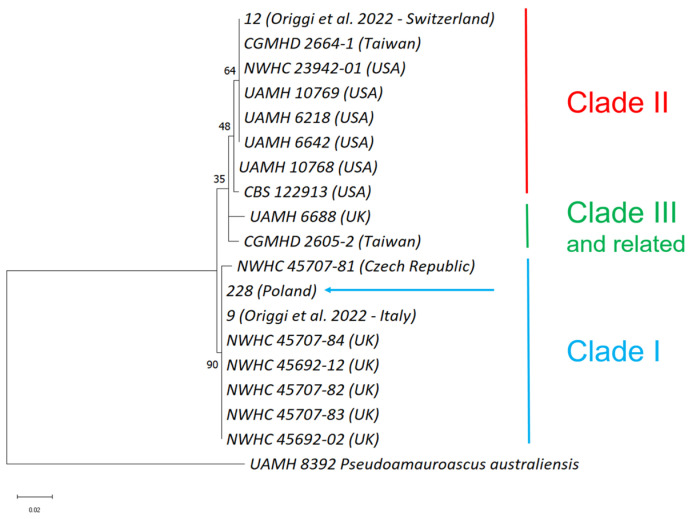
The maximum likelihood phylogenetic tree obtained from three concatenated partial regions (ACT, TEF, and ITS2) of *O. ophidiicola* sequences from this study (sample ID 228, arrow), GenBank, and the literature (Table S4). The blue line, red line, and green line highlight strains belonging to Clade I, II, and III (and related), respectively, as described by [30,31,32]. Sample ID 228 clusters with Clade I sequences. Sequences belonging to Clade II or Clade III (and related) are arranged in agreement with their lineage identity. Bootstrap support values are represented at nodes. *Pseudoamauroascus australiensis* was used to root the tree.

qPCR products from ID 149 (ITS2), 218 (nad1), and 228 (ITS2 and nad1) and conventional PCR products from 228 were submitted to Sanger sequencing. Sequences from qPCR targeting ITS2 of 149 and nad1 of 218 showed 100% identity with *O. ophidiicola* sequences in BLAST, while sequences from qPCR of 228 were incomplete (88% identity and 10 gaps each—Table S3). Amplicons from conventional PCRs (ACT, TEF, ITS2, and ITS2L) of 228 shared 100% identity with *O. ophidiicola* sequences in BLAST. Sample ID 228 was the only one submitted to clade characterisation. Subsequently to concatenation, alignment, trimming, and a second concatenation, the software-computed sequences were in total 341 bp long (i.e., 92 bp ACT, 158 bp TEF, and 91 bp ITS2—see Table S3). The resulting maximum likelihood phylogenetic tree is represented in Figure 4.

**Figure 5:**
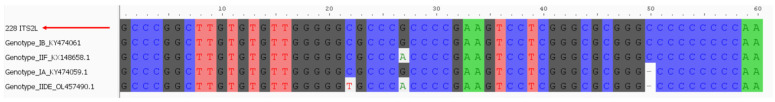
Alignment of the ITS2 sequence from sample ID 228 with four representative ITS2 region *O. ophidiicola* genotype sequences according to Blanvillain et al. [41]. Consensus nucleotides (filled characters) and different single-nucleotide polymorphisms (SNPs—blank characters) compared to 228 (arrow) are highlighted. *O. ophidiicola* from 228 belongs to the genotype I-B, being identical to KY47406.

The ITS2L sequence of sample ID 228 was aligned with representative *O. ophidiicola* genotype sequences deposited in GenBank to differentiate *O. ophidiicola* clades according to Blanvillain et al. [41]. The AliView alignment is shown in Figure 5, while the output of BLAST pairwise alignment is reported in Table S5. ID 228 showed 100% identity and no gaps with NWHC 45707-81 (KY474061—an isolate from Czechia), belonging to genotype I-B according to Blanvillain et al. [41].

Positive samples belonged to two specimens of *Z. longissimus* (149, 218) and one of *N. natrix* (228). The prevalence for *Z. longissimus* was 3.7% (2/54, 95% CI [0.0%, 9.3%]), and 20% (1/5, 95% CI [0.0%, 60.0%]) for *N. natrix*, while for both species combined, it was 5.1% (3/59, 95% CI [0.0%, 11.9%]). *O. ophidiicola* detections came from specimens collected during August 2011 (149: *Z. longissimus*), July 2012 (218: *Z. longissimus*), and August 2012 (228: *N. natrix*) (see Figure S3). Only one moult out of three that tested positive for *O. ophidiicola* showed gross signs consistent with ophidiomycosis (i.e., sample ID 218—see Figure S4).

The statistical analysis revealed no significant dependence between pairs of categorical variables including *O. ophidiicola* detection, time of year (month), species, and absence/presence of gross signs, as indicated by Chi-square tests (see Table S6). The logistic regression analysis also supported these findings, showing no significant influence of the month or clinical signs on the likelihood of testing positive for the fungus. However, there was a marginally significant association between species and *O. ophidiicola* detection (*p* = 0.0724; see Table S6). 

## 4. Discussion

The findings from this study provide insights into the historical prevalence of *Ophidiomyces ophidiicola* on the ophidiofauna from the San River Valley in the Bieszczady Mountains, Poland. The results are pivotal given the scarcity of data on the occurrence of *O. ophidiicola* in isolated snake populations and its implications for conservation efforts. Moreover, it leverages the use of archived and museal samples to give insights on ophidiomycosis in a local context.

*Ophidiomyces ophidiicola* has occurred in wild snakes living in the Bieszczady region at least since 2011. Taking into account the diversity of sample size per species, the total prevalence of *O. ophidiicola* resulting from the analysed archived moults (5.1%) appears to be consistent with the findings coming from decade-newer samples from the same study site (9.8%, Blanvillain et al. [41]). Furthermore, the same genotype I-B has been detected, confirming that it was not recently introduced. Given the higher prevalence found in *N. natrix* compared to *Z. longissimus*, in both our study (grass snake: 20%; Aesculapian snake: 3.7%) and Blanvillain et al.’s work [41] (grass snake: 40%; Aesculapian snake: 2.4%), it is plausible that in this location, grass snakes are the main reservoirs and shedders of *O. ophidiicola*. In fact, logistic regression indicated a less-than-significant association between *O. ophidiicola*-positive detection and species. It is important to note that the unbalanced categories of some variables within the samples—only 3 out of 59 samples tested positive for the pathogen, and only 5 samples belonged to *N. natrix*—likely limit the statistical power of the analysis, potentially obscuring true associations. Moreover, it cannot be ruled out whether any of the collected moults originated from the same individual, and any “recapture” data cannot be excluded in the analysis. Nonetheless, our data are in line with other European studies highlighting the greater tropism of the fungus to semi-aquatic snakes and the *Natrix* genus [38,41].

The first clinical ophidiomycosis lesions can be observed 8 days after experimental inoculation [19]. The lack of gross skin lesions on some moults despite the detection of *O. ophidiicola* DNA may be a consequence of an initial stage of fungal infection (preclinical infection) at the time of moulting, a subclinical infection, a clearance of a minor superficial infection by shedding, or environmental contamination [12,38]. This indicates that the absence of noticeable skin lesions does not equate to the absence of the fungus. On the other hand, the association of cutaneous lesions with molecular detection is highly suggestive of cutaneous ophidiomycosis (i.e., apparent ophidiomycosis—[12,26,36]). However, since the macroscopic tegumentary signs of ophidiomycosis are nonspecific, it cannot be excluded that they were caused by other etiologic agents (see [12,13,38]).

Our sampling could have yielded some false negatives because we sampled only three pieces of moults (plus one additional if gross signs were detected) to avoid destroying the entire specimen and to facilitate convenient transportation. However, grinding the sheds into powder before DNA extraction (see [42]) should mitigate this issue by ensuring a more homogeneous sample and increasing the likelihood of detecting the target DNA. 

This study was carried out on archived specimens, where DNA could be more fragmented compared to fresh samples. Indeed, two out of three positive samples yield a successful detection in only one of the two qPCR targets (ITS2 or nad1) likely due to DNA fragmentation, poor DNA extraction, or low fungal loads. For sample ID 149 and 218, the Sanger sequencing from the selected qPCR wells—showing melting peaks/temperatures and positive calls at End RFUs consistent with a positive detection—was fundamental for the confirmation of results, since the samples did not give any other specific amplification. The use of two independent genomic and mitochondrial *O. ophidiicola* qPCR targets permitted a broader range of detection, reducing the risk of false negative results and giving complementary information. Therefore, the use of more than one target seems crucial for retrospective studies on *O. ophidiicola*. In such cases, implementing an internal control PCR for host DNA detection could help to assess the quality of preserved nucleic acids and the reliability of the results (see [40]).

The ITS2 SYBR Green-based qPCR coupled with ITS2L conventional PCR shows promising potential. Sequencing ITS2L amplicons is an effective method to validate ITS2 qPCR positive results and simultaneously characterise the fungus. In Marini et al.’s work [36], all SYBR Green-based qPCR positive results were submitted to direct sequencing of the qPCR amplicons to provide a proof of concept of the reliability of the method and to confirm the probe sequence designed for the original TaqMan real-time PCR by Bohuski et al. [68]. However, in Marini et al.’s work [36], some of the qPCR sequences showed one or two gaps, due to the potential low-quality outcome of Sanger sequencing when targeting amplicons < 100 bp (see [70]). With the ITS2L assay, this problem was overcome when the amplification of the conventional PCR reaction occurred as in sample ID 228. Nonetheless, the ITS2L assay, as in the ITS2, ACT, and TEF assays according to Origgi et al. [33], was not able to amplify *O. ophidiicola* DNA from sample ID 149 and 218. Thus, this approach can be hindered by the fragmentation of poor-quality nucleic acids, but its reliability would likely be higher in a prospective study where fresh samples with less DNA degradation are expected. Furthermore, the ITS2L assay can yield results comparable to the amplification used by Blanvillain et al. [41] and Origgi et al. [33], given that all the SNPs used to characterise the genotypes are detectable in the sequence (see Figure 1, Tables S3 and S5). The original nested PCR by Blanvillain et al. [41] used the panfungal primer ITS3 and ITS4 in the first reaction, and ITS3 with Oo-rt-ITS-R (referred to in this text also as Bohuski RV—see Figure 1 and Table S2) in the second reaction. The *O. ophidiicola*-specific primers (Origgi FW and Bohuski RV) used in ITS2L permit an amplification of a shorter product (c.a. 100 bp shorter) and should be more specific compared to the second reaction of the nested PCR used by Blanvillain et al. [41]. The use of the *O. ophidiicola*-specific primers of ITS2L or ITS2 primers by Origgi et al. [33] in a nested PCR assay might enhance both sensitivity and specificity toward *O. ophidiicola* in the case of genotyping. Moreover, the reduced length of the amplicons can be particularly useful for old and museal samples with potentially fragmented DNA (see [33]).

In this study, the clade characterisation via the employment of the maximum likelihood phylogenetic tree used a concatenation of partial sequences of ACT, TEF, and ITS2, totalling approximately 350 bp in length. These sequences feature notable SNPs crucial for *O. ophidiicola* characterisation, as identified by Origgi et al. ([33]—see Table S3), who employed them in constructing a single phylogenetic tree per target. Despite the use of shorter sequences, our findings remain consistent with the previous literature [30,31,32]. While this method may provide less detailed resolution compared to phylogenetic analyses utilising longer sequences, it appears to be effective to simultaneously use multiple *O. ophidiicola* DNA regions for comparing different lineages. However, it should be noted that this method was not capable of clustering subclades within the identified clades, both deleting or including gaps.

The *O. ophidiicola* genotype I-B from our study, dating back to 2012, was also the only subclade detected from the same area in the prospective study by Blanvillain et al. [41]. While no *O. ophidiicola* from Clade II was identified in the Bieszczady region, its presence is known to significantly increase the probability of infection in *Z. longissimus* and is likely responsible for more severe disease manifestations compared to Clade I [41]. This is further supported by a case of Aesculapian snake mortality in the Iberian Peninsula, likely due to ophidiomycosis caused by the II-D/E lineage [43]. The detection of Clade II-D/E across Europe, particularly in nearby Czechia and Ukraine [41], should be viewed as a significant menace of pathogen pollution in the San River Valley. Preventing the potential spillover of this strain is essential, especially in areas where *Z. longissimus* is threatened, like the San River Valley where nesting sites are limited and the snake population is dense.

In particular, limited nesting sites create challenges for females of this group-living sedentary species [62], forcing them to migrate in search of new sites [56]. This migration exposes females (and their unlaid oviductal eggs) to increased predation risk. Another strategy is utilising collective egg-laying sites with other females [52,71]. *O. ophidiicola* has demonstrated the ability to survive in natural soils, with Campbell et al. [17] finding a higher prevalence of the fungus in hibernacula soils compared to topsoils. This suggests that breeding mounds and hibernacula may serve as environmental reservoirs for *O. ophidiicola*, which may persist in its viable forms [17,72,73]. Consequently, 2–3 decades ago, when breeding microhabitats were scarce in the San River Valley, *O. ophidiicola* could have expanded its range and hosts in the area through breeding sites. The scarcity of these sites likely concentrated snake populations, potentially allowing a single infected female to transmit *O. ophidiicola* to numerous other females and their hatchlings. This scenario underscores the importance of active conservation through habitat manipulation, such as constructing artificial egg-laying sites to reduce female density per site and potentially impede pathogen spread. While the exact origin of *O. ophidiicola* in this region remains uncertain, as it could have been naturally present for centuries, its introduction into this Polish population might also be linked to herpetoculturists’ interest in Aesculapian snakes. Numerous documented cases of deliberate snake translocations through pseudo-conservation activities exist [62]. Additionally, individual *Z. longissimus* or *N. natrix* specimens were occasionally collected in San River Valley, kept in captivity for several years, and subsequently released [74], potentially facilitating *O. ophidiicola* spillover from captive-bred or wild-caught individuals.

Although the magnitude of ophidiomycosis impact on wild snake populations remains controversial (see [12]), these findings emphasise the importance of ongoing surveillance and targeted conservation efforts to prevent or mitigate *O. ophidiicola* effects, particularly in regions where snake habitats are limited. Effective habitat management and creation are necessary to reduce snake density and prevent pathogen transmission. Implementing improved DNA extraction methods and exploring the potential to cultivate and isolate the fungus, along with updated population estimates for the current decade, are advised to better understand the contemporary situation in the San River Valley. Additionally, considering the scarcity of screening initiatives for reptiles and most amphibians in Poland and the limited studies on fungal pathogens [41,75], the investigation on other mycotic agents, such as *Paranannizziopsis* sp. (see [76]), is important to safeguard ophidiofauna. These future perspectives are crucial for developing effective conservation strategies and ensuring the long-term survival of vulnerable snake populations in the Bieszczady Mountains.

## 5. Conclusions

In conclusion, testing fungal pathogens in exuvial material has confirmed the historical presence of *O. ophidiicola* in Bieszczady Mountains, which has only recently been discovered through a prospective study [41]. The employed methodology enabled the investigation of pathogen prevalence in archived samples at relatively low costs (see [36]). This approach can provide critical insights into the spread of EIDs in reptile populations over time.

Our findings suggest that *O. ophidiicola* has been present in the Bieszczady ophidian population for a considerable period. This long-term presence of the pathogen could be a contributing factor to the observed low population numbers within the main last remaining Polish site of *Zamenis longissimus*. Nonetheless, even if the prevalence of our work is comparable with the prospective study by Blanvillain et al. [41], we are not able to conclude whether Clade I-B concurs for a potential lower fitness of *Z. longissimus* in the studied area since population estimations for the current decade are missing. Therefore, further investigations on *Ophidiomyces* and population size are urgently needed to understand potential mechanisms of host–pathogen interaction within Aesculapian snakes in San River Valley. Understanding the historical prevalence and impact of *O. ophidiicola* is vital for the conservation and management of reptile populations, emphasising the importance of continued surveillance and research on reptile pathogens.

## Figures and Tables

**Table 1:** Specimens sampled and analysed in this study. Subsequent number, IOP (internal) sample ID, species, location of finding, coordinates of finding, date of finding (yyyymm), and absence or presence of gross signs are reported.

Number	Sample ID	Species	Location	Coordinates	Date	Gross Signs
1	20	Zl	NA	NA	200407	y
2	21	Zl	Koliba	49.26, 22.51	201008	n
3	22	Nn	NA	NA	NA	n
4	23	Zl	Zatwarnica	49.23, 22.56	201008	y
5	25	Zl	Kamieniołom	49.25, 22.53	201008	y
6	26	Zl	Zatwarnica	49.23, 22.56	201008	y
7	28	Zl	NA	NA	201008	n
8	50	Nn	Tartak	49.21, 22.55	201009	n
9	52	Zl	Koliba	49.26, 22.51	201109	n
10	53	Nn	NA	NA	NA	n
11	59	Nn	NA	NA	NA	n
12	60	Zl	Kopiec Żaka	49.23, 22.55	201109	y
13	65	Zl	Druga pasieka	49.26, 22.50	201107	n
14	66	Zl	Druga pasieka	49.26, 22.50	201107	y
15	69	Zl	Zatwarnica	49.22, 22.55	201107	y
16	70	Zl	Zakole Sanu	49.25, 22.54	201107	y
17	71	Zl	Koliba	49.26, 22.51	201107	n
18	94	Zl	Zatwarnica	49.22, 22.55	201106	n
19	95	Zl	Zatwarnica	49.22, 22.55	201106	n
20	106	Zl	Sękowiec	49.23, 22.56	201107	n
21	107	Zl	Zatwarnica	49.22, 22.55	201107	n
22	108	Zl	Zatwarnica	49.22, 22.55	201107	n
23	109	Zl	Zatwarnica	49.22, 22.55	201107	n
24	111	Zl	Zatwarnica	49.22, 22.55	201107	n
25	122	Zl	Koliba	49.26, 22.51	201107	n
26	123	Zl	Koliba	49.26, 22.51	201107	n
27	124	Zl	Koliba	49.26, 22.51	201107	y
28	125	Zl	Koliba	49.26, 22.51	201107	n
29	126	Zl	Koliba	49.26, 22.51	201107	n
30	127	Zl	Zatwarnica	49.22, 22.55	201107	n
31	136	Zl	Sękowiec	NA	201107	y
32	141	Zl	Zatwarnica	49.22, 22.55	201107	n
33	142	Zl	Zatwarnica	49.22, 22.55	201107	y
34	144	Zl	Dwernik	49.22, 22.62	201108	y
35	145	Zl	Dwernik	49.22, 22.62	201108	y
36	147	Zl	Zatwarnica	49.22, 22.55	201108	n
37	149	Zl	Sękowiec	49.23, 22.56	201108	n
38	155	Zl	Dwernik	49.22, 22.62	201108	n
39	175	Zl	Dwernik	49.22, 22.62	201108	n
40	179	Zl	Koliba	49.26, 22.51	201108	n
41	181	Zl	Koliba	49.26, 22.51	201108	n
42	182	Zl	Koliba	49.26, 22.51	201108	n
43	186	Zl	Kamieniołom	49.25, 22.53	201108	n
44	187	Zl	Koliba	49.26, 22.51	201108	n
45	190	Zl	Zatwarnica	49.22, 22.55	201105	n
46	191	Zl	Zatwarnica	49.22, 22.55	201105	n
47	215	Zl	Zatwarnica	49.22, 22.55	201208	y
48	216	Zl	Koliba	49.26, 22.51	201205	n
49	218	Zl	Dwernik	49.22, 22.62	201207	y
50	223	Zl	Zatwarnica	49.22, 22.55	201206	n
51	224	Zl	Koliba	49.26, 22.51	201207	n
52	225	Zl	Zatwarnica	49.22, 22.55	201207	n
53	226	Zl	Zatwarnica	49.22, 22.55	201208	y
54	227	Zl	Zatwarnica	49.22, 22.55	201208	y
55	228	Nn	Tworylne	49.26, 22.47	201208	n
56	229	Zl	Dwernik	49.22, 22.62	201209	n
57	230	Zl	Dwernik	49.22, 22.62	201209	n
58	231	Zl	Zatwarnica	49.22, 22.55	NA	n
59	Martwy ^1^	Zl	Zatwarnica	49.23, 22.55	201205	n

^1^ Road-killed specimen. Zl—*Zamenis longissimus*, Nn—*Natrix natrix*, NA—not assigned; n—no gross signs, y—visible gross signs.

## Data Availability

Data generated or analysed during this study are partially included in this published article and its supplementary information files. Raw data generated during and/or analysed during the current study are available from the corresponding authors on reasonable request.

## Supplementary Materials

The following supporting information can be downloaded at: https://www.mdpi.com/article/10.3390/microorganisms12071467/s1, Figure S1: Representative Zamenis longissimus moults considered with gross signs consistent with *O. ophidiicola* infection (brownish crusts, dysecdysis). A: Sample ID 66. B: Sample ID 142; Figure S2: Bio-Rad CFX Maestro software showing the ITS2 and nad1 amplification curve (above) and melting curves and peaks (below) of the samples considered positive for one (149: ITS2; 218: nad1) or both targets (228). Note the positive controls. The qPCR run was performed with the same plate and cycling conditions; Figure S3: Graphs showing species and months of shed collection, with *O. ophidiicola* detection outcomes. A: The histogram showing the total number of *Zamenis longissimus* (grey bars) and *Natrix natrix* (green bars) sheds collected during different months in the study period. Positive *O. ophidiicola* detections are represented by darker shades. B: The bubble plot showing species and months of sheds collected. Circle size is dependent on the sample number (see legend), with red circles representing positive samples. This plot also includes sheds with unknown collection times; Figure S4: The distribution of snake samples based on the presence of gross signs and molecular test results. The histogram shows the number of samples (*y*-axis) categorised by the presence or absence of gross signs (*x*-axis). Each category is further divided into positive (red) and negative (grey) test results for *O. ophidiicola* presence; Table S1: Concentrations (ng/µL) of DNA extracted from each sample measured with TECAN Spark^®^ Multimode Microplate Reader. Extracted DNA in bold yielded positive results in SYBR Green-based qPCR for ITS2, and underlined ones for nad1 (see Table S3). All samples were exuviae except one marked with “*” (tissue from a road-killed specimen); Table S2: PCR types and primers used in this study; Table S3: Sequences obtained from successful amplifications after End RFU, melting curves/temperatures, or gel inspection. Primer sequences or their reverse complements are not excised from the whole sequences and are highlighted in bold. The sequence underlined in the ITS2 sequences corresponds to the probe marked with 6-carboxyfluorescein (FAM) and Black Hole Quencher^®^-1 (BHQ-1) used during TaqMan qPCR (5′-(FAM)TCTCGCTCGAAGACCCGATCG(BHQ-1)-3′—Bohuski et al. [68]); nucleotides within the probe highlighted in red are missing. Nucleotides highlighted in pale blue are single-nucleotide polymorphisms (SNPs) differentiating lineages *sensu* Origgi et al. [33] and, just for ITS2 region, Blanvillain et al. [41]. Nucleotides highlighted in green are the additional ITS2 SNPs differentiating genotypes *sensu* Blanvillain et al. [41]; Table S4: *Ophidiomyces ophidiicola* sequences used for the phylogenetic analysis. GenBank accession numbers or literature references for the DNA regions utilised are provided. *Pseudoamauroascus australiensis* was used as an outgroup; Table S5: Output of the BLAST alignment (view: pairwise with dots for identities) of representative *O. ophidiicola* genotype sequences deposited in GenBank with 228 ITS2L sequence. *O. ophidiicola* genotypes are differentiated according to Blanvillain et al., 2024 [41]; Table S6: Details of the statistical analysis.
