# Supplementary material for: Retrospective Detection of Ophidiomyces ophidiicola from Snake Moults Collected in Bieszczady Mountains, Poland
Source: Microorganisms. 2024 Jul 19;12(7):1467. doi: 10.3390/microorganisms12071467 (PMC11279008; doi:10.3390/microorganisms12071467)
Supplement: Supplementary file 1 [file microorganisms-12-01467-s001.zip › microorganisms-3108565-supplementary.pdf]

Supplementary Files

Retrospective detection of *Ophidiomyces ophidiicola* from snake moults collected in Bieszczady mountains, Poland

Daniele Marini [1,2], Piotr Szczygieł [3], Katarzyna Kurek [4], Matteo R. Di Nicola [5,6,7,*], Jean-Lou C.M. Dorne [8], Maria Luisa Marenzoni [2], Joëlle Rüegg [1], Stanisław Bury [9,10], Łukasz Kiraga [11]

Affiliations:

[1] Department of Organismal Biology, Evolutionary Biology Centre, Uppsala University, 75236 Uppsala, Sweden; daniele.marini@ebc.uu.se; joelle.ruegg@ebc.uu.se

[2] Department of Veterinary Medicine, University of Perugia, 06126 Perugia, Italy; daniele.marini@dottorandi.unipg.it ; marialuisa.marenzoni@unipg.it

[3] Scientific Society of Veterinary Medicine Students, Faculty of Veterinary Medicine, Warsaw University of Life Sciences—SGGW, 02-776 Warsaw, Poland; s202489@sggw.edu.pl (P.S.)

[4] Department of Wildlife Conservation, Institute of Nature Conservation Polish Academy of Science, 31-120 Cracow, Poland; kkurek@iop.krakow.pl

[5] Faculty of Veterinary Medicine, Department of Pathobiology, Pharmacology and Zoological Medicine, Wildlife Health Ghent, Ghent University, 9820 Merelbeke, Belgium; matteodinicola86@libero.it

[6] Unit of Dermatology and Cosmetology, IRCCS San Raffaele Hospital, 20132 Milan, Italy
[7] Asociación Herpetológica Española, 28911 Leganés, Spain

[8] Methodology and Scientific Support Unit, European Food Safety Authority (EFSA), Parma 43126, Italy; Jean-Lou.DORNE@efsa.europa.eu

[9] Department of Comparative Anatomy, Institute of Zoology and Biomedical Research, Jagiellonian University, 30-387 Cracow, Poland; stanislaw.bury@uj.edu.pl

[10] NATRIX Herpetological Association, Wroclaw, Poland

[11] Division of Pharmacology and Toxicology, Department of Preclinical Sciences, Institute of Veterinary Medicine, Warsaw University of Life Sciences—SGGW, 02-786 Warsaw, Poland; lukasz_kiraga@sggw.edu.pl

*Correspondence: matteodinicola86@libero.it

Figure S1: Representative *Zamenis longissimus* moults considered with gross signs consistent with *O. ophidiicola* infection (brownish crusts, dysecdysis). A: Sample ID 66. B: Sample ID 142. Photocredit: Daniele Marini.

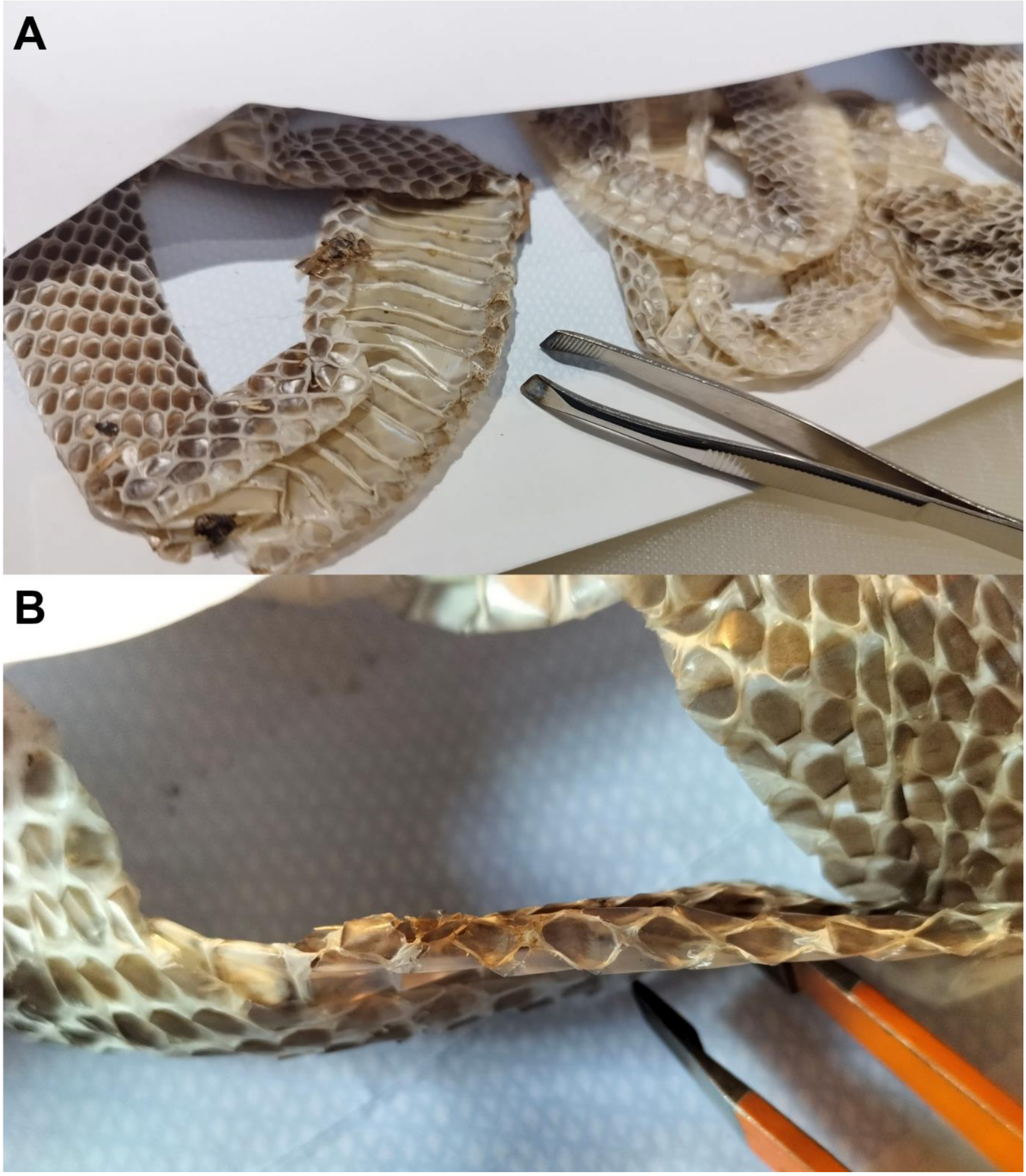

Figure S2: Bio-Rad CFX Maestro software showing ITS2 and nad1 amplification curve (above) and melting curves and peaks (below) of the samples considered positives for one (149: ITS2; 218: nad1) or both targets (228). Note the positive controls. The qPCR run was done with the same plate and cycling conditions.

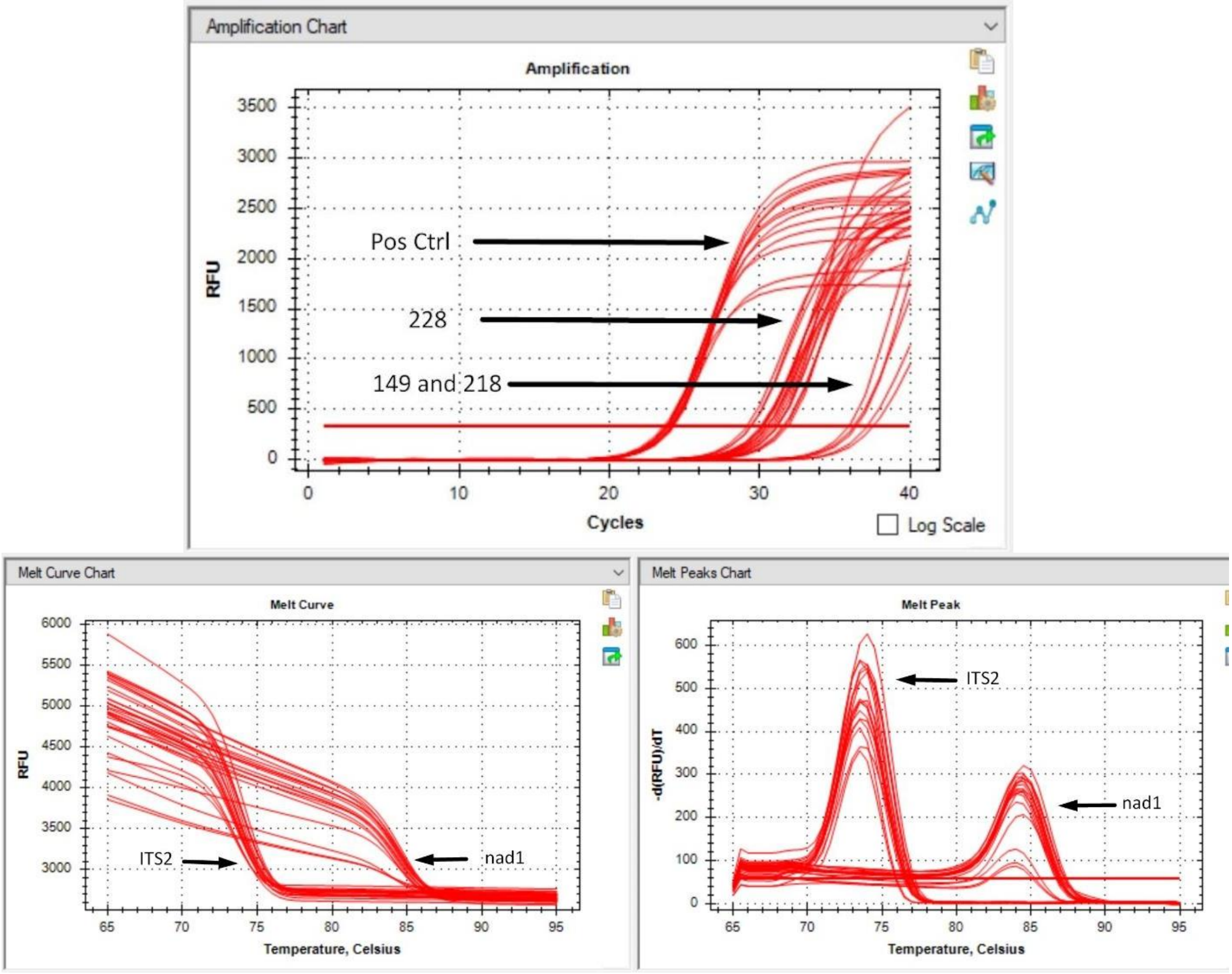

Figure S3: Graphs showing species and months of shed collection, with *O. ophidiicola* detection outcomes. A: Histogram showing the total number of *Zamenis longissimus* (grey bars) and *Natrix natrix* (green bars) sheds collected during different months in the study period. Positive *O. ophidiicola* detections are represented by darker shades. B: Bubble plot showing species and months of sheds collected. Circle size is dependent on the sample number (see legend), with red circles representing positive samples. This plot also includes sheds with unknown collection times.

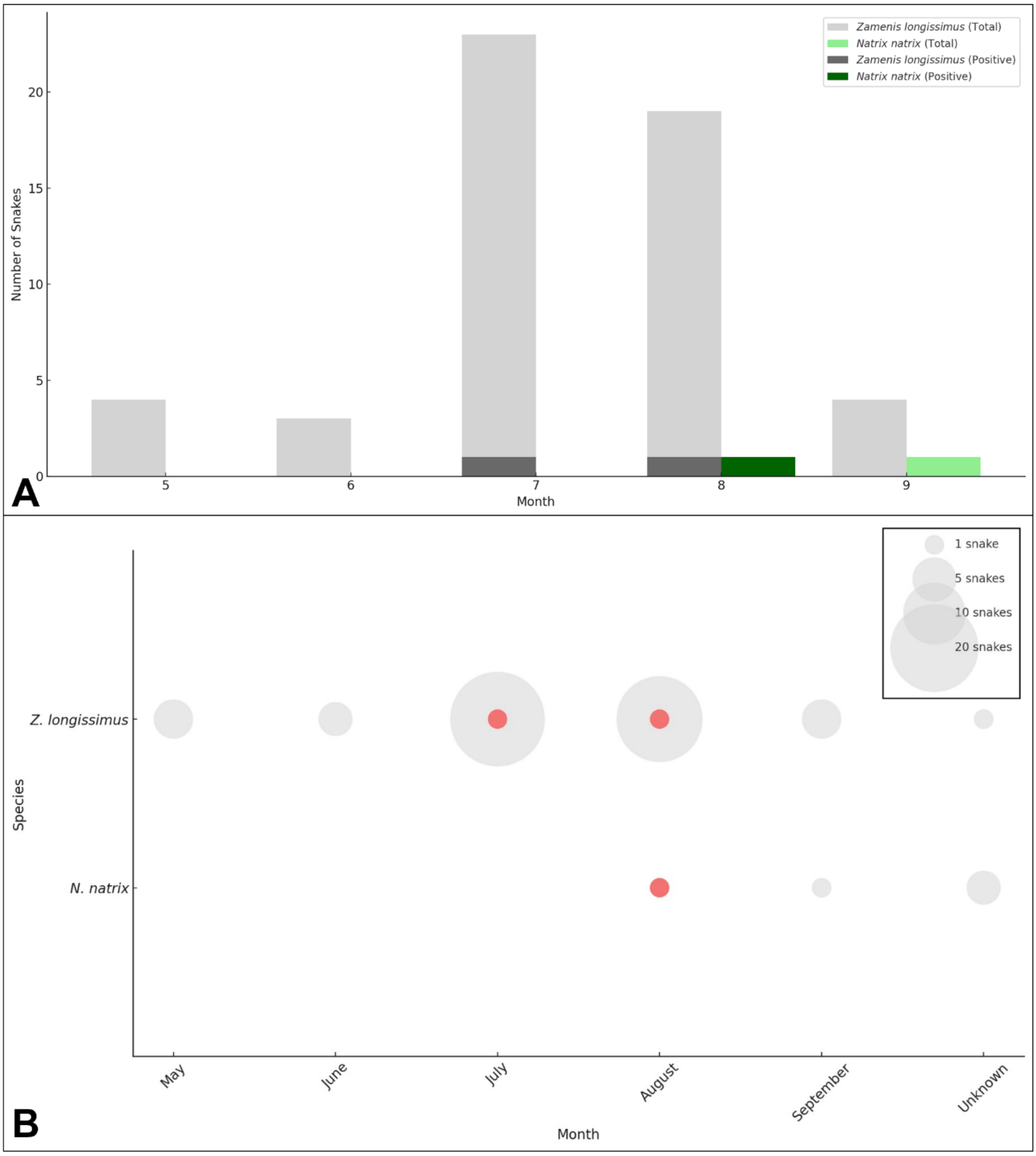

Figure S4: Distribution of snake samples based on the presence of gross signs and molecular test results. The histogram shows the number of samples (y-axis) categorised by the presence or absence of gross signs (x-axis). Each category is further divided into positive (red) and negative (grey) test results for *O. ophidiicola* presence.

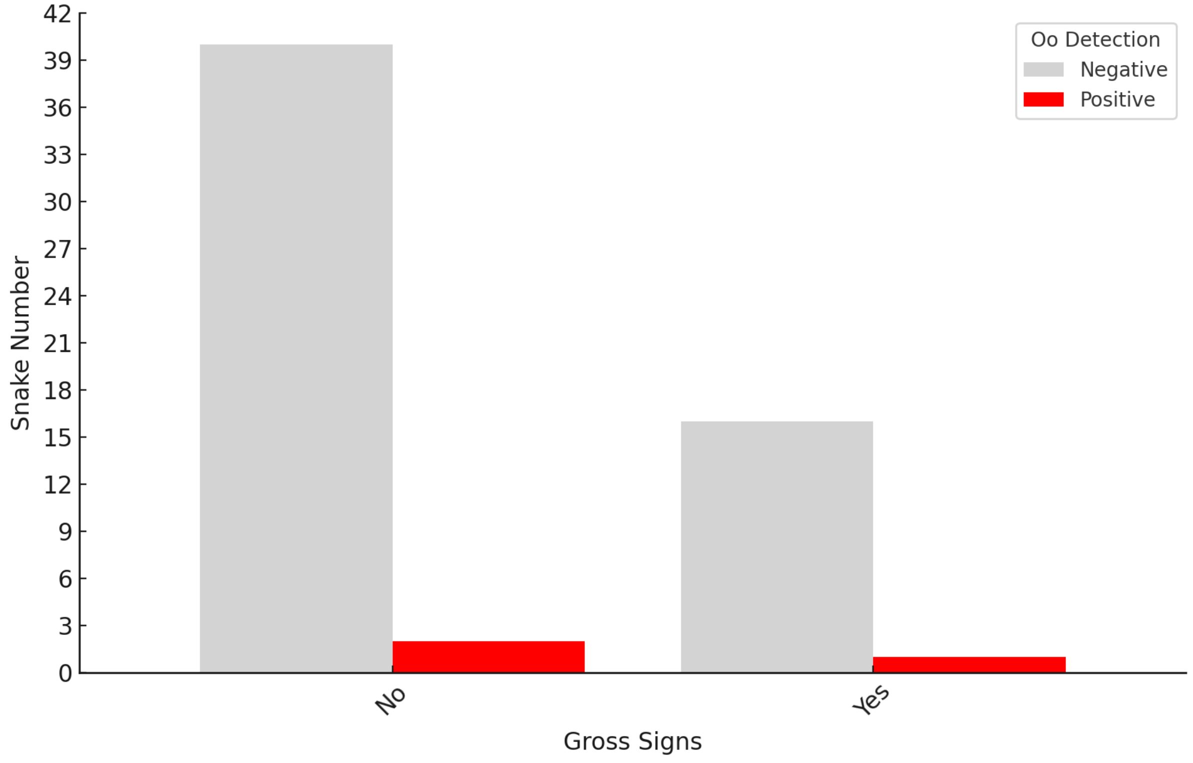

Table S1: Concentrations (ng/µl) of DNA extracted from each sample measured with TECAN Spark® Multimode Microplate Reader. Extracted DNA in bold yielded positive results at SYBR Green-based qPCR for ITS2, and underlined ones for nad1 (see Table S3). All samples were exuviae except one marked with "*" (tissue from a road-killed specimen).

Order	Sample ID	ng/µl	Order	Sample ID	ng/µl
1	20	395,47	30	127	232,11
2	21	135,27	31	136	865,84
3	22	215,2	32	141	98,51
4	23	762,36	33	142	23,48
5	25	164,54	34	144	111,48
6	26	344,95	35	145	70,04
7	28	757,08	36	147	17,45
8	50	243,46	37	**149**	**44,87**
9	52	230,71	38	155	73,5
10	53	271,43	39	175	63,11
11	59	196,95	40	179	328,52
12	60	167,67	41	181	283,88
13	65	727,08	42	182	280,2
14	66	612,91	43	186	199,16
15	69	1272,16	44	187	66,44
16	70	1047,82	45	190	351,27
17	71	733,14	46	191	186,32
18	94	96,93	47	215	1427,26
19	95	399,98	48	216	445,18
20	106	681,63	49	218	772,24
21	107	125,47	50	223	103,37
22	108	59,97	51	224	297,32
23	109	78,71	52	225	266,99
24	111	23,82	53	226	335,87
25	122	470,24	54	227	211,88
26	123	584,35	55	**228**	**363,11**
27	124	542,49	56	229	487,56
28	125	122,24	57	230	125,65
29	126	112,63	58	231	143,62
			59	MARTWY*	1319,63

Table S2: PCR types and primers used in this study.

**PCR type**	**Region**	**Primers**	**Amplicon size (bp) comprising primers**	**Primers reference**
qPCR (SYBR Green-based)	Internal transcribed spacer 2 (ITS2 within the rRNA gene complex [5.8 S – 28S] - genomic)	Oo-rt-ITS-F:<br>5′ - GAGTGTATGGGAATCTGTTTC - 3′<br>Oo-rt-ITS-R:<br>5′ - GGTCAAACCGGAAAGAATG - 3′	82	Bohuski E, Lorch JM, Griffin KM, Blehert DS (2015). TaqMan real-time polymerase chain reaction for detection of *Ophidiomyces ophiodiicola*, the fungus associated with snake fungal disease. BMC Vet Res 11(1), 1-10. https://doi.org/10.1186/s12917-015-0407-8
qPCR (SYBR Green-based)	NADH dehydrogenase subunit 1 (nad1 - mitochondrial)	Oo-nad1-F:<br>5′ - ACTTGATTGTTTCTCTAGTC - 3′<br>Oo-nad1-R:<br>5′ - AGGGAAAGAAGCTCTAAC - 3′	85	Lorch, JM, Price SJ, Lankton JS, Drayer AN (2021) Appendix 2. Confirmed cases of Ophidiomycosis in Museum specimens from as early as 1945, United States. Emerg Infect Dis 27. https://doi.org/10.3201/eid2707.204864
Conventional PCR	Internal transcribed spacer 2 (ITS2 within the rRNA gene complex [5.8 S – 28S] - genomic)	OoPhyl_ITS_ Origg_F:<br>5′-TGTCCGAGCGTCATTGCAACC-3′<br>OoPhyl_ITS_ Origg_R:<br>5′-AACAGATTCCCATACACTCAGACACC-3′	137-138	Origgi, FC. Pisano, SR, Glaizot, O, Hertwig, ST, Schmitz, A, Ursenbacher, S (2022) *Ophidiomyces ophiodiicola*, Etiologic Agent of Snake Fungal Disease, in Europe since Late 1950s. . Emerg Infect Dis 28. https://doi.org/10.3201/eid2810.220564
Conventional PCR	Actin gene (ACT - genomic)	OoPhyl_ACT_ Origg_F:<br>5′-TTAGATTTCCAGCAAGAGATCCAGACTG-3′<br>OoPhyl_ACT_ Origg_R:<br>5′-CCAAGACGCTGGGTTGGAAAAG-3′	142	Origgi, FC. Pisano, SR, Glaizot, O, Hertwig, ST, Schmitz, A, Ursenbacher, S (2022) *Ophidiomyces ophiodiicola*, Etiologic Agent of Snake Fungal Disease, in Europe since Late 1950s. . Emerg Infect Dis 28. https://doi.org/10.3201/eid2810.220564
Conventional PCR	Transcription elongation factor 1 α (TEF - genomic)	OoPhyl_TEF_ Origg_F:<br>5′-CCAGCCCAACTATCAAACTTTGGC-3′<br>OoPhyl_TEF_ Origg_R:<br>5′-TGATACCACGCTCACGCTCGG-3′	203	Origgi, FC. Pisano, SR, Glaizot, O, Hertwig, ST, Schmitz, A, Ursenbacher, S (2022) *Ophidiomyces ophiodiicola*, Etiologic Agent of Snake Fungal Disease, in Europe since Late 1950s. . Emerg Infect Dis 28. https://doi.org/10.3201/eid2810.220564
Conventional PCR	Internal transcribed spacer 2 (ITS2 within the rRNA gene complex [5.8 S – 28S] - genomic)	OoPhyl_ITS_ Origg_F:<br>5′-TGTCCGAGCGTCATTGCAACC-3′<br>Oo-rt-ITS-R:<br>5′ - GGTCAAACCGGAAAGAATG - 3′	200-201	Origgi et al. 2022 & Bohuski et al. 2015 (see above).<br>Called ITS2 Longer (ITS2L – see Figure 1)

Table S3: Sequences obtained from successful amplifications after End RFU, melting curves/temperatures or gel inspection. Primer sequences or their reverse complements are not excised from the whole sequences and are highlighted in bold. The sequence underlined in the ITS2 sequences corresponds to the probe marked with 6-carboxyfluorescein (FAM) and Black Hole Quencher®-1 (BHQ-1) used during TaqMan qPCR (5′-(FAM)TCTCGCTCGAAGACCCGATCG(BHQ-1)-3′ - Bohuski *et al.* [68]); nucleotides within the probe highlighted in red are missing. Nucleotides highlighted in pale blue are single nucleotide polymorphisms (SNPs) differentiating lineages *sensu* Origgi *et al.* [33] and, just for ITS2 region, Blanvillain *et al.* [41]. Nucleotides highlighted in green are the additional ITS2 SNPs differentiating genotypes *sensu* Blanvillain *et al.* [41].

**Sample**	**Oo DNA target region**	**PCR type and primers used**	**Obtained sequence (5'-3'; strand plus/plus)**	**Percent identity (BLAST) with first(s) Oo sequence(s) in GenBank (in square brackets)**
149	ITS2	SYBR green based qPCR, Bohuski et al. 2015	**GAGTGTATGGGAATCTGTTTC**TGTCTCGCT<br>CGAAGACCCGATCGGCGCCCGTCGTCAACC<br>CCC**CATTCTTTCCGGTTTGACC**	100% (82/82) [KF225599.1]
218	nad1	SYBR green based qPCR, Lorch et al. 2021	**ACTTGATTGTTTCTCTAGTC**TTAATTTAGC<br>TtTAAAAACTAGTTTTCTTATCTTTGTTTT<br>TATTTGA**GTTAGAGCTTCTTTCCCT**	100% (85/85) [NC_082836.1]
228	ITS2	SYBR green based qPCR, Bohuski et al. 2015	**GAGTGTATGGGAATCTGTTTc**TGTCTCGCT<br>CGAAGACCCGATC----------CGTC-<br>ACCCCC**CATTCTTTCCGGTTTGACC**	88% (72/82; 10/82 gaps) [OQ612704.1 ; KF225599.1]
	nad1	SYBR green based qPCR, Lorch et al. 2021	**ACTTGATTGTTTCTCTAGTC**TTAATTTAGC<br>TTTAA----------<br>TCTTATCTTTGTTTTTATTTGA**GTTAGAGC**<br>**TTCTTTCC--**	88% (73/83; 10/83 gaps)) [NC_082836.1]
	ITS2	Conventional PCR, Origgi et al. 2022	**TGTCCGAGCGTCATTGCAACC**CCCTCAAGC<br>CCGGCTTGTGTGTTGGGGGCGCCCGCCCCG<br>AAGTCCTCGGGCGCGGGCCCCCCCCCAAAT<br>GCAGTGGCGGCACCGAGTTCCT**GGTGTCTG**<br>**AGTGTATGGGAATCTGTT**	100% (138/138) [KY474061.1]
	ACT	Conventional PCR, Origgi et al. 2022	**TTAGATTTCCAGCAAGAGATCCAGACTG**CT<br>GCTCAGAGCTCTAGCTTGGAAAGATCTTAT<br>GAGCTTCCTGACGGCCAAGTCATTACCATT<br>GGCAACGAGCGATTCCGTGCTCCCGAAGCC<br>**CTTTTCCAACCCAGCGTCTTGG**	100% (142/142) [KY474070.1]
	TEF	Conventional PCR, Origgi et al. 2022	**CCAGCCCAACTATCAAACTTTGGC**AGAATT<br>GTCGATCTTTGACCAATCATGCCTGACCCC<br>TTTGAACCATGCATTTTTTACCTTGACGCT<br>CTTCAGTATACTAATATGTTTCCCCCCTTA<br>GGAAGCCGAAGAGTTGGGCAAGAAATCCTT<br>CAAATATGCCTGGGTTCTTGACAAATTGAA<br>GG**CCGAGCGTGAGCGTGGTATCA**	100% (203/203) [KY474085.1]

	ITS2L	Conventional PCR, FW Origgi et al. 2022, RV Bohuski et al. 2015	**TGTCCGAGCGTCATTGCAACC**CCCTCAAGC<br>CCGGCTTGTGTGTTGGGGGCGCCCGCCCCG<br>AAGTCCTCGGGCGCGGGCCCCCCCCCAAAT<br>GCAGTGGCGGCACCGAGTTCCTGGTGTCTG<br>AGTGTATGGGAATCTGTTTCTGTCTCGCTC<br>GAAGACCCGATCGGCGCCCGTCGTCAACCC<br>CC**CATTCTTTCCGGTTTGACC**	99% (200/201) [KF225599.1]<br><br>100% (196/196) [KY474061.1]
	ACT + TEF + ITS2	Trimmed and concatenated ACT, TEF and ITS2 used for phylogenetic analysis<br><br>Total: 341 bp	CTGCTCAGAGCTCTAGCTTGGAAAGATCTT<br>ATGAGCTTCCTGACGGCCAAGTCATTACCA<br>TTGGCAACGAGCGATTCCGTGCTCCCGAAG<br>CCAGAATTGTCGATCTTTGACCAATCATGC<br>CTGACCCCTTTGAACCATGCATTTTTTACC<br>TTGACGCTCTTCAGTATACTAATATGTTTC<br>CCCCCTTAGGAAGCCGAAGAGTTGGGCAAG<br>AAATCCTTCAAATATGCCTGGGTTCTTGAC<br>AAATTGAAGGCCCTCAAGCCCGGCTTGTGT<br>GTTGGGGGCGCCCGCCCCGAAGTCCTCGGG<br>CGCGGGCCCCCCCCCAAATGCAGTGGCGGC<br>ACCGAGTTCCT	/

Table S4: *Ophidiomyces ophidiicola* sequences used for the phylogenetic analysis. GenBank accession numbers or literature references for the DNA regions utilised are provided. *Pseudoamauroascus australiensis* was used as an outgroup.

Isolate or Sequence Number	Fungal Species	Country	GenBank accession numbers or other sources		
			ACT	TEF	ITS
9	Oo	Italy	Origgi et al. 2022<br>DOI: https://doi.org/10.3201/eid2810.220564		
12	Oo	Switzerland	Origgi et al. 2022<br>DOI: https://doi.org/10.3201/eid2810.220564		
CBS 122913	Oo	USA	HF547891	KY474079	EU715819
CGMHD 2605-2	Oo	Taiwan	MT912507	MT912511	MT905070
CGMHD 2664-1	Oo	Taiwan	MT912509	MT912513	MT906449
NWHC 23942-01	Oo	USA	KY474076	KY474091	KY474065
NWHC 45692-02	Oo	UK	KY474070	KY474085	KY474059
NWHC 45692-12	Oo	UK	KY474071	KY474086	KY474060
NWHC 45707-81	Oo	Czech Republic	KY474072	KY474087	KY474061
NWHC 45707-82	Oo	UK	KY474073	KY474088	KY474062
NWHC 45707-83	Oo	UK	KY474074	KY474089	KY474063
NWHC 45707-84	Oo	UK	KY474075	KY474090	KY474064
UAMH 10768	Oo	USA	KY474068	KY474083	KF477234
UAMH 10769	Oo	USA	KY474069	KY474084	KF477235
UAMH 6218	Oo	USA	KY474066	KY474080	KF477227
UAMH 6642	Oo	USA	KY474067	KY474081	KC884267
UAMH 6688	Oo	UK	KY474078	KY474082	KF477228
UAMH 8392	*P. australiensis*	Australia	KY474077	KY474092	AJ131787

Table S5: Output of the BLAST alignment (view: pairwise with dots for identities) of representative *O. ophidiicola* genotypes sequences deposited in GenBank with 228 ITS2L sequence. Oo genotypes are differentiated according to Blanvillain *et al*. [41].

Genotype_IB_KY474061

```
Query: Ophidiomyces ophiodiicola voucher NWHC 45707-81 small subunit ribosomal RNA gene, partial
sequence; internal transcribed spacer 1 and 5.8S ribosomal RNA gene, complete sequence; and internal
transcribed spacer 2, partial sequence Query ID: KY474061.1 Length: 611


>
Sequence ID: Query_3767233 Length: 201
Range 1: 1 to 196

Score:363 bits(196), Expect:6e-105,
Identities:196/196(100%),  Gaps:0/196(0%), Strand: Plus/Plus

Query  416  TGTCCGAGCGTCATTGCAACCCCCTCAAGCCCGGCTTGTGTGTTGGGGGCGCCCGCCCCG  475
Sbjct  1    ............................................................  60

Query  476  AAGTCCTCGGGCGCGGGccccccccAAATGCAGTGGCGGCACCGAGTTCCTGGTGTCTG  535
Sbjct  61   ............................................................  120

Query  536  AGTGTATGGGAATCTGTTTCTGTCTCGCTCGAAGACCCGATCGGCGCCCGTCGTCAACCC  595
Sbjct  121  ............................................................  180

Query  596  CCCATTCTTTCCGGTT  611
Sbjct  181  ................  196
```

Genotype_IA_KY474059.1

```
Query: Ophidiomyces ophiodiicola voucher NWHC 45692-02 small subunit ribosomal RNA gene, partial
sequence; internal transcribed spacer 1 and 5.8S ribosomal RNA gene, complete sequence; and internal
transcribed spacer 2, partial sequence Query ID: KY474059.1 Length: 610


>
Sequence ID: Query_7219227 Length: 201
Range 1: 1 to 196

Score:355 bits(192), Expect:1e-102,
Identities:195/196(99%),  Gaps:1/196(0%), Strand: Plus/Plus

Query  416  TGTCCGAGCGTCATTGCAACCCCCTCAAGCCCGGCTTGTGTGTTGGGGGCGCCCGCCCCG  475
Sbjct  1    ............................................................  60

Query  476  AAGTCCTCGGGCGCGGG-ccccccccAAATGCAGTGGCGGCACCGAGTTCCTGGTGTCTG  534
Sbjct  61   .................C..........................................  120

Query  535  AGTGTATGGGAATCTGTTTCTGTCTCGCTCGAAGACCCGATCGGCGCCCGTCGTCAACCC  594
Sbjct  121  ............................................................  180

Query  595  CCCATTCTTTCCGGTT  610
Sbjct  181  ................  196
```

Genotype_IIF_KX148658.1

```
Query: Ophidiomyces ophidiicola strain NWHC 24281-01-04-01 18S ribosomal RNA gene, partial sequence;
internal transcribed spacer 1 and 5.8S ribosomal RNA gene, complete sequence; and internal transcribed
spacer 2, partial sequence Query ID: KX148658.1 Length: 611


>
Sequence ID: Query_7294243 Length: 201
```

```
Range 1: 1 to 196

Score:357 bits(193), Expect:3e-103,
Identities:195/196(99%),  Gaps:0/196(0%), Strand: Plus/Plus

Query  416  TGTCCGAGCGTCATTGCAACCCCCTCAAGCCCGGCTTGTGTGTTGGGGGCGCCCACCCCG  475
Sbjct  1    ......................................................G.....  60

Query  476  AAGTCCTCGGGCGCGGGccccccccAAATGCAGTGGCGGCACCGAGTTCCTGGTGTCTG  535
Sbjct  61   ............................................................  120

Query  536  AGTGTATGGGAATCTGTTTCTGTCTCGCTCGAAGACCCGATCGGCGCCCGTCGTCAACCC  595
Sbjct  121  ............................................................  180

Query  596  CCCATTCTTTCCGGTT  611
Sbjct  181  ................  196
```

Genotype_IIDE_OL457490.1

```
Query: Ophidiomyces ophidiicola strain NWHC 27242-2 small subunit ribosomal RNA gene, partial
sequence; internal transcribed spacer 1 and 5.8S ribosomal RNA gene, complete sequence; and internal
transcribed spacer 2, partial sequence Query ID: OL457490.1 Length: 610


>
Sequence ID: Query_7333147 Length: 201
Range 1: 1 to 196

Score:344 bits(186), Expect:2e-99,
Identities:193/196(98%),  Gaps:1/196(0%), Strand: Plus/Plus

Query  416  TGTCCGAGCGTCATTGCAACCCCCTCAAGCCCGGCTTGTGTGTTGGGGGTGCCCACCCCG  475
Sbjct  1    .................................................C....G.....  60

Query  476  AAGTCCTCGGGCGCGGG-cccccccAAATGCAGTGGCGGCACCGAGTTCCTGGTGTCTG  534
Sbjct  61   .................C..........................................  120

Query  535  AGTGTATGGGAATCTGTTTCTGTCTCGCTCGAAGACCCGATCGGCGCCCGTCGTCAACCC  594
Sbjct  121  ............................................................  180

Query  595  CCCATTCTTTCCGGTT  610
Sbjct  181  ................  196
```

Table S6: Details of the statistical analysis.

Chi-Square Tests for Independence

Variable Pair	χ^2	df	p-value
Oo detection and Species	1.54	1	0.215
Oo detection y and Period of Year	1.55	4	0.818
Oo detection and Gross Signs	0.00	1	1.000
Gross Signs and Species	0.034	1	0.854
Gross Signs and Period	4.35	4	0.361

Logistic Regression Analysis

Variable	Coefficient	p-value	95% CI (Lower)	95% CI (Upper)
Intercept	-2.4301	0.6622	-13.3329	8.4728
Month	-0.1590	0.8390	-1.6926	1.3746
Species	3.7812	0.0724	-0.3434	7.9057
Gross Signs	0.8597	0.5688	-2.0977	3.8171
